# Transcription factor EB-mediated mesenchymal stem cell therapy induces autophagy and alleviates spinocerebellar ataxia type 3 defects in neuronal cells model

**DOI:** 10.1038/s41419-022-05085-0

**Published:** 2022-07-18

**Authors:** Xiaobo Han, Jean de Dieu Habimana, Amy L. Li, Rongqi Huang, Omar Mukama, Weiyue Deng, Ling Wang, Yuying Zhang, Wei Wang, Sihao Deng, Kexin Peng, Bin Ni, Shusheng Zhang, Jufang Huang, Xiao-xin Yan, Zhiyuan Li

**Affiliations:** 1 CAS Key Laboratory of Regenerative Biology, Guangdong Provincial Key Laboratory of Stem Cell and Regenerative Medicine, Guangzhou Institutes of Biomedicine and Health, Chinese Academy of Sciences, Guangzhou, China; 2 Department of Anatomy and Neurobiology, Xiangya School of Medicine, Central South University, Changsha, China; 3 NHC Key Laboratory of Birth Defect for Research and Prevention, Hunan Provincial Maternal and Child Health Care Hospital, Changsha, China; 4 Changsha Stomatological Hospital, Changsha, China; 5 Bioland Laboratory, Guangzhou, China; 6 GZMU-GIBH Joint School of Life Sciences, Guangzhou Medical University, Guangzhou, China; 7 GIBH-HKU Guangdong-Hong Kong Stem Cell and Regenerative Medicine Research Centre, GIBH-CUHK Joint Research Laboratory on Stem Cell and Regenerative Medicine, Guangzhou, China

**Keywords:** Spinocerebellar ataxia, Neurodegeneration

## Abstract

Defects in ataxin-3 proteins and CAG repeat expansions in its coding gene ATXN3 cause Spinocerebellar Ataxia Type 3 (SCA3) or Machado-Joseph disease (MJD) polyglutamine neurodegenerative disease. The mutant proteins aggregate as inclusion bodies in cells and compete with wild-type ataxin-3, which leads to neuronal dysfunction or death and impairs Beclin1-mediated autophagy. It has been reported that Mesenchymal stem cells (MSCs) can reliably treat several neurodegenerative diseases. Herein, we used a Transcription Factor EB (TFEB) nuclear translocation-mediated MSCs co-culture approach to reconstitute autophagy and lysosomal biogenesis, and reduce SCA3-like behaviors in induced pluripotent stem cells (iPSCs)-derived neuron cells models. Our iPSCs model showed enhanced expression of autophagy proteins, attenuated the expression and toxic effects of mutant ataxin-3 on neurons, and alleviated the effects of ataxin-3 on autophagy. Therefore, MSCs are associated with autophagy-inducing therapy and compared to animal models, our MSCs co-culture could be used as a novel and potential therapeutic approach to study SCA3 disease and other neurodegenerative diseases.

## Introduction

Spinocerebellar Ataxia type 3 (SCA3), also known as Machado-Joseph disease (MJD), is an autosomal dominant inherited neurodegenerative disease. SCA3 is reported to be the most prevalent subtype of all SCAs, second to Huntington’s disease (HD) [1, 2]. The main symptoms of SCA3 are progressive ataxia due to cerebellar and brainstem dysfunction, accompanied by pyramidal and extrapyramidal symptoms, amyotrophy, and fasciculations [3, 4]. As one of the nine polyglutamine neurodegenerative diseases, SCA3 is caused by an abnormally expanded polyglutamine (polyQ) repeat in the ataxin-3 protein. PolyQ proteins aggregate and form intracellular inclusions bodies in the neurons resulting in the dysfunction and degeneration of neurons [5].

Autophagy is a highly conserved lysosome-mediated catabolic process involved in the degradation of damaged organelles or toxic protein aggregates to preserve cellular homeostasis [6]. In the past decade, significant progress has been made in understanding the molecular mechanisms, regulation, and effects of autophagy on physiology and pathophysiology, including infections, cancer, neurodegeneration, cardiovascular disorders, and aging [7–9]. It is also necessary for neuronal development and axon homeostasis [10]. The autophagosome-lysosomal pathway degrades aged organelles and aggregated proteins to maintain neuronal homeostasis, which is especially important in neurodegenerative diseases. There is mounting evidence that autophagy is impaired in neurodegenerative diseases such as amyotrophic lateral sclerosis (ALS), Alzheimer’s disease (AD) [11, 12], Parkinson’s disease (PD), and HD [13, 14]. Moreover, several reports revealed a link between autophagy-lysosome dysfunction and neurodegenerative diseases. Therefore, inducing neuronal autophagy is crucial for the treatment of neurodegeneration [15–17].

The transcription factor EB (TFEB) is one of the major transcriptional regulators of autophagy and promotes the expression of genes for autophagosome formation and lysosomal biogenesis [18]. Reddy et al., reported that TFEB is highly expressed in the central nervous system [19]. Autophagy dysfunction is a defining characteristic of almost all neurodegenerative diseases. As such, TFEB dysfunction is associated with the pathogenesis of many neurodegenerative diseases, including AD, PD, HD, and SCA3, and TFEB overexpression can halt their progression [20–23]. Therefore, the induction of TFEB activity represents a potential therapeutic strategy for human neurodegenerative diseases.

Cell therapy for treating neurodegenerative diseases has been around for decades with varying success [24]. Mesenchymal stem cells (MSCs) are pluripotent stem cells that have increasingly been shown to treat a variety of neurological diseases by secreting neurotrophic growth factors [25, 26], attenuating neuroinflammation[27, 28], or releasing extracellular vesicles such as exosomes [29, 30]. Therefore, herein, we used rare genetic patient iPSCs as disease cells model to explore the central neural system (CNS) degeneration and our results showed that iPSCs fate processing was altered by TFEB-mediated MSCs, which regulated target cell autophagy and reduced SCA3-like biochemical deficits. Therefore, this autophagy-mediated MSCs treatment could provide a broader prospect for mesenchymal stem cell therapy [31].

## Results

### Establishment of iPSCs-derived neuron models and identification of MSCs and exosomes

**Fig. 1:**
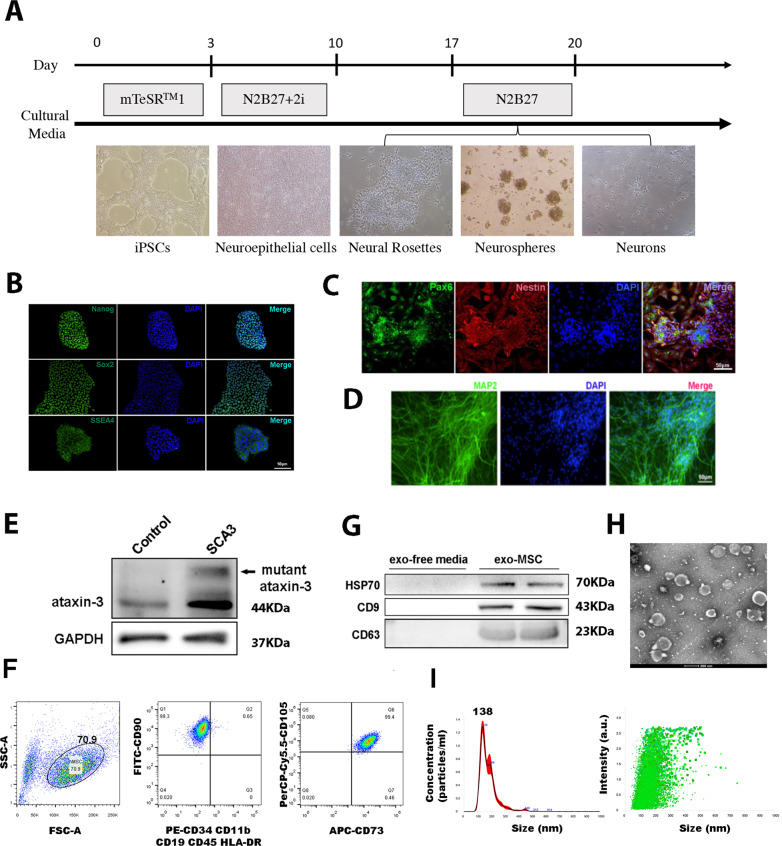
The establishment of iPSCs-derived neuron cell models in SCA3 patients and the identification of MSCs and exosomes. **A** Time diagram of induction of iPSCs to neurons. **B** Immunofluorescence analysis of iPSCs related markers (scale bar: 50 μm). **C** NSCs related markers identification (scale bar: 50 μm). **D** NCs related marker MAP2 analysis (scale bar: 50 μm). **E** mutant Ataxin-3 protein identification in neurons. **F** MSCs related markers were identified by flow analysis, MSCs expressed highly CD90, CD107, and CD73. **G** Western blotting analysis of exosome-specific markers CD63, CD9, and HSP70. **H** Transmission electron microscopy of exosomes (scale bar: 200 μm). **I** Nanoparticle tracking analysis of exosomes.

First, we induced neurons from iPSCs under various culture medium (Fig. 1A). The karyotype analysis confirmed the patients’ urine-derived iPSCs maintained normal karyotype after induction (Fig. S1A). Immunofluorescence analysis showed iPSCs highly expressed pluripotency-related genes Nanog, Sox2, and SSEA4 (Fig. 1B) consistent with flow cytometry, and RT-qPCR results (S1B, C). Moreover, the teratoma formation experiments proved that iPSCs had the differentiation ability into three layers (Fig. S1D).

The obtained patient’s iPSCs were induced into neurons. As shown in Fig. 1C, neural stem cell markers Pax6 and Nestin were highly expressed and displayed neural rosettes. Similarly, Map2 and Beta-tubulin neuronal cell markers were also highly expressed (Fig. 1D and S1E), confirming successful differentiation into neurons. These neurons were characterized by cellular alternation associated with significant expression of mutant ataxin-3 confirmed by our model SCA3 patient-derived iPSCs (Fig. 1E).

We next elucidated the exosome presence in our umbilical cord blood-derived MSCs. As shown in Fig. 1F, the MSCs positively expressed CD90, CD107, and CD73, while negatively expressed CD34, CD11b, CD19, and CD45 surface markers, and obtained vesicles via supercentrifugation highly expressed the exosome-specific markers CD63, CD9, and HSP70 (Fig. 1G). The transmission electron microscopy also showed a large number of bilayer membrane vesicles (Fig. 1H) and nanoparticle tracking analysis showed vesicles concentrated at about 138 nm (Fig. 1I), indicating the presence of exosomal vesicles.

### MSCs therapy alleviated the toxic effects of mutant proteins on neurons by increasing autophagy flux in neuronal cells of SCA3 patients

To verify the effect of MSCs treatment, SCA3 patient-derived neurons were treated with MSCs and MSC supernatant-containing exosome inhibitor GW4869. It was evident through the CCK8 assay that the survival and proliferation of patients’ cells were significantly increased in the MSCs-treated group compared to the inhibitor control (Fig. S2A). The release of lactate dehydrogenase (LDH) is associated with apoptosis or necrosis. Therefore, we measured LDH levels in the cell medium, which decreased after MSCs treatment and increased with MSCs-containing GW4869 treatment (Fig. S2B). Similar results were obtained for apoptosis-related proteins Bcl-2 and Bax. The Bcl-2 decreased after treatment, while the expression of anti-apoptotic protein Bax increased (Fig. S2C). The detection of neuronal cells growth showed more axons and dendrites for efficient signal transmission (Fig. S2D).

**Fig. 2:**
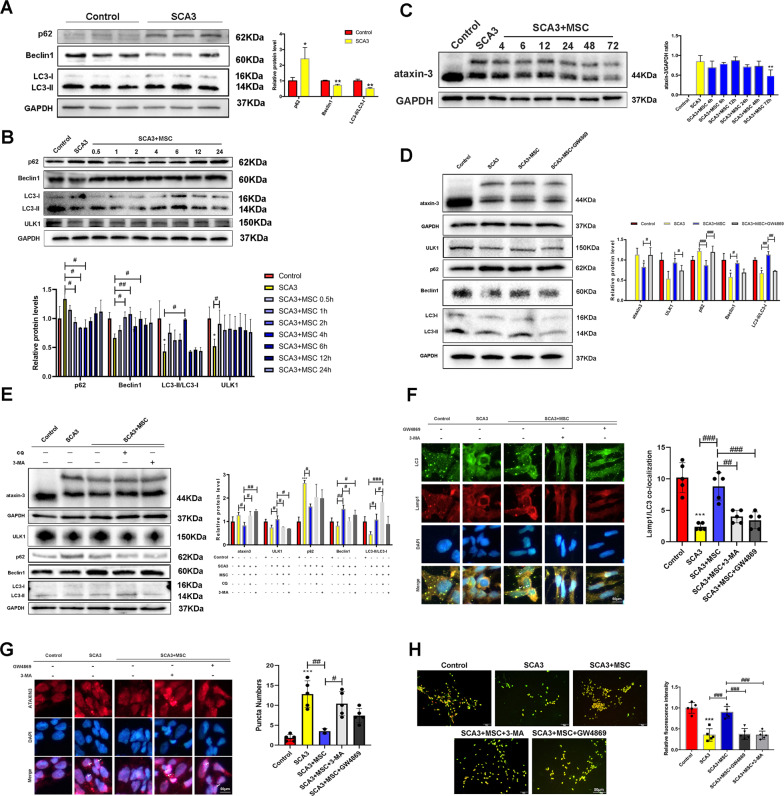
MSCs reduce intracellular mutant protein ataxin-3 levels in SCA3 patients. **A** Westen blotting detection of autophagy-related proteins in Control and SCA3 groups. **B** Detection of p62, Beclin1, ULK1, and LC3 at different treatment times after MSCs-SCA3 patient co-culture for 1 h. **C** Time-dependent expression of mutant ataxin-3 after MSCs therapy for 72 h. **D** The effects of GW4869 on the expression of p62, Beclin1, LC3, ULK1, and ataxin-3. **E** The effects of 3-MA and CQ on the expression of p62, Beclin1, LC3, ULK1, and ataxin-3 after MSCs therapy. **F** LC3 and Lamp1 co-localization after MSCs by immunofluorescence (scale bar: 50 μm). **G** DAPI and mutant ataxin-3 co-localization after MSCs by immunofluorescence (scale bar: 50 μm). **H** Acidic vesicles induced by MSCs treatment using acridine orange staining (scale bar: 50 μm). *p < 0.05, **p < 0.01, ***p < 0.001 vs Control group, ^#^*p* < 0.05, ^##^*p* < 0.01, ^###^*p* < 0.001.

Notably, autophagy proteins in neurons of SCA3 patients were significantly lower than those of normal controls (Fig. 2A). After MSCs-SCA3 patients’ cells co-culture, ULK1, Beclin1 and LC3-II were significantly increased, and p62 was significantly decreased in the patient group. Intriguingly, the MSCs co-culture decreased significantly mutant ataxin-3 levels (Fig. 2C). The increased autophagic flux was significantly reversed after the addition of MSC supernatant-containing exosome inhibitor GW4869 (Fig. 2D). Similarly, autophagy flux decreased after treatment with autophagy inhibitor 3-MA, Chloroquine (CQ) and GW4869. In contrast, the expression of LC3-II was significantly enhanced after CQ treatment (Fig. 2E). Moreover, immunofluorescence showed detectable autophagic proteins LC3 and Lamp1 and a large number of autophagosomes in the MSCs group (Fig. 2F), and the level of ataxin-3 protein in the nucleus decreased significantly (Fig. 2G), while more acidic vesicles were observed (Fig. 2H). Thus, the autophagy flux reduced the level of mutant ataxin-3 protein in SCA3 neurons attributable to MCSs treatment.

### MSCs induced TFEB nuclear translocation in neuronal cells of SCA3 patients

**Fig. 3:**
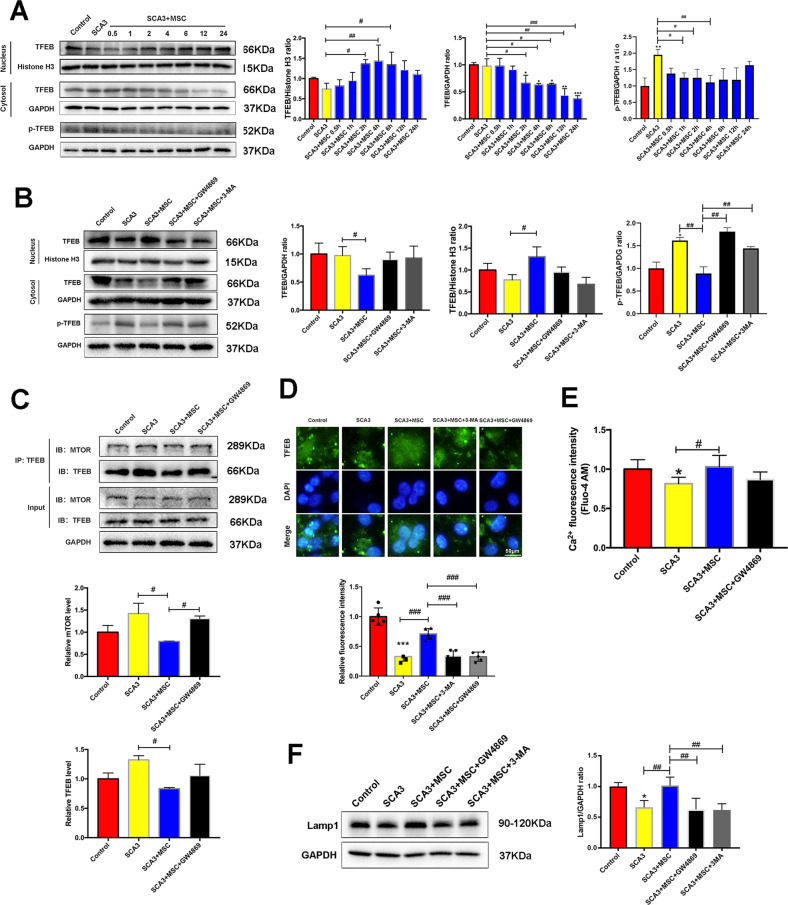
MSCs induced TFEB nuclear translocation in neuronal cells of SCA3 patients. **A** The expression of TFEB and p-TFEB at different treatment times in the cytoplasm and nucleus after MSCs therapy. **B** The effects of GW4869 and 3-MA on the expression of TFEB and p-TFEB in the cytoplasm and nucleus after MSCs therapy. **C** Co-IP assay for mTORC1-TFEB interaction. **D** TFEB and DAPI co-localization after MSCs by immunofluorescence (scale bar: 50 μm). **E** The detection of Lysosomal Ca^2+^ release into the cytoplasm after MSCs therapy. **F** The expression levels of Lamp1. *p < 0.05, **p < 0.005, ***p < 0.001 vs Control group, ^#^*p* < 0.05, ^##^*p* < 0.01, ^###^*p* < 0.001.

To examine whether the TFEB, an autophagy regulator, is involved, MSCs-SCA3 patients were co-cultured and compared with 3-MA and GW4869 inhibitors-treated groups. TFEB nuclear translocation increased significantly in MSCs-treated SCA3 patients and increased significantly in a time-dependent manner (Fig. 3A, B, D). The finding that mTORC1 phosphorylates TFEB, and this interaction isolates TFEB in the cytoplasm prompted us to detect mTORC1-TFEB interaction [32]. Our Co-immunoprecipitation (Co-IP) and western blotting data suggested that MSCs treatment decreases mTORC1-TFEB interaction and dephosphorylation of TFEB (Fig. 3B, C). Moreover, as TFEB nuclear dephosphorylation is accompanied by Ca^2+^ release from the lysosome to the cytoplasm, we detected Ca^2+^ levels in the cytoplasm. It was found that Ca^2+^ levels significantly increased in the cytoplasm after MSCs treatment (Fig. 3E). Furthermore, we also detected lysosomal protein after MSCs co-culture. As predicted, MSCs treatment promoted the expression of lysosomal protein Lamp1 (Fig. 3F). Overall, we revealed that MSCs-SCA3 neurons co-culture induced TFEB nuclear translocation in SCA3 neurons based on TFEB nuclear dephosphorylation and mTORC1 dissociation, Ca^2+^ flux, and Lamp1-mediated lysosomal biogenesis.

### MSCs co-culture activated autophagolysosome and alleviated mutant ataxin-3 protein

**Fig. 4:**
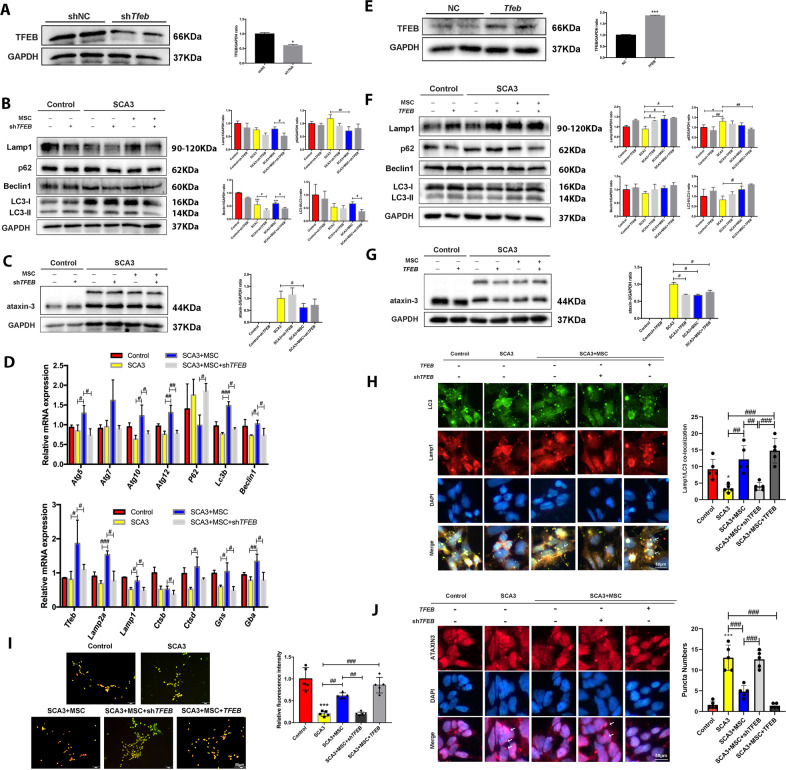
MSCs co-culture activated autophagolysosome and alleviated mutant ataxin-3 protein-dependent on *TFEB* nuclear translocation in SCA3 neurons. **A** The expression of TFEB after adding Lentiviral *shTFEB* plasmid. **B** The effects of *shTFEB* on the expression of p62, Beclin1, LC3, and lamp1 after MSCs therapy. **C** The effects of *shTFEB* on the expression of mutant ataxin-3 after MSCs therapy. **D** The mRNA expression of autophagy and lysosome-related genes. **E** The expression of TFEB after adding Lentiviral *TFEB* plasmid. **F** The effects of *TFEB* on the expression of p62, Beclin1, LC3, and lamp1 after MSCs therapy. **G** The effects of *TFEB* on the expression of mutant ataxin-3 after MSCs therapy. **H** LC3 and Lamp1 co-localization after *shTFEB* and *TFEB* by immunofluorescence (scale bar: 50 μm). **I** The effects of *shTFEB* and *TFEB* on the acidic vesicles after MSCs treatment (scale bar: 50 μm). **J** DAPI and mutant ataxin-3 co-localization after *shTFEB* and *TFEB* by immunofluorescence (scale bar: 50 μm). *p < 0.05, ***p < 0.001 vs Control group, ^#^*p* < 0.05, ^##^*p* < 0.01^, ###^*p* < 0.001.

To further evaluate the effects of the *TFEB* gene on autophagy and mutant ataxin-3 protein, we knocked down the *TFEB* gene expression in neurons (Fig. 4A and S3A) and validated the effects of shRNA for TFEB (*shTFEB*) after co-culture with MSCs. The *shTFEB* reduced the levels of intracellular autophagic proteins Lamp1, Beclin1, and LC3-II while increasing the levels of the intracellular mutant ataxin-3 protein (Fig. 4A, B). We also observed a significant increase of autophagy and lysosomal-related genes in MSCs-treated SCA3 neurons and reduced significantly p-mTOR levels, however, *shTFEB* addition reversed this reduction (Fig. 4B and S3C). Intriguingly, the *TFEB*-overexpression changed autophagic and mutant ataxin-3 protein levels (Fig. S3B) with a significant increase in autophagy flux and a further reduction in the expression of ataxin-3 (Fig. 3D, E). Simultaneously, more acidic vesicles and lysosomes appeared after MSCs treatment, which significantly decreased with *shTFEB* addition and increased with *TFEB* overexpression (Fig. 3G and S3D).

The immunofluorescence detection of autophagic proteins LC3 and Lamp1 and mutant ataxin-3 after *shTFEB* and *TFEB* showed that *shTFEB* inhibited the increase of autophagosomes and a decrease of ataxin-3 protein after MSCs treatment. After overexpressing *TFEB*, we also observed increased co-localization between LC3 and Lamp1 after *shTFEB* and *TFEB*, depicting that the number of intracellular autophagosomes increased while the mutant proteins entered the nucleus decreased (Fig. 3F, H). Therefore, the *TFEB* gene regulates MSCs-induced autophagy-lysosome pathway and promotes mutant ataxin-3 removal.

### MSCs treatment activated autophagy by regulating the AKT/mTOR and AMPK/mTOR signaling pathways to reduce the intracellular mutant ataxin-3

**Fig. 5:**
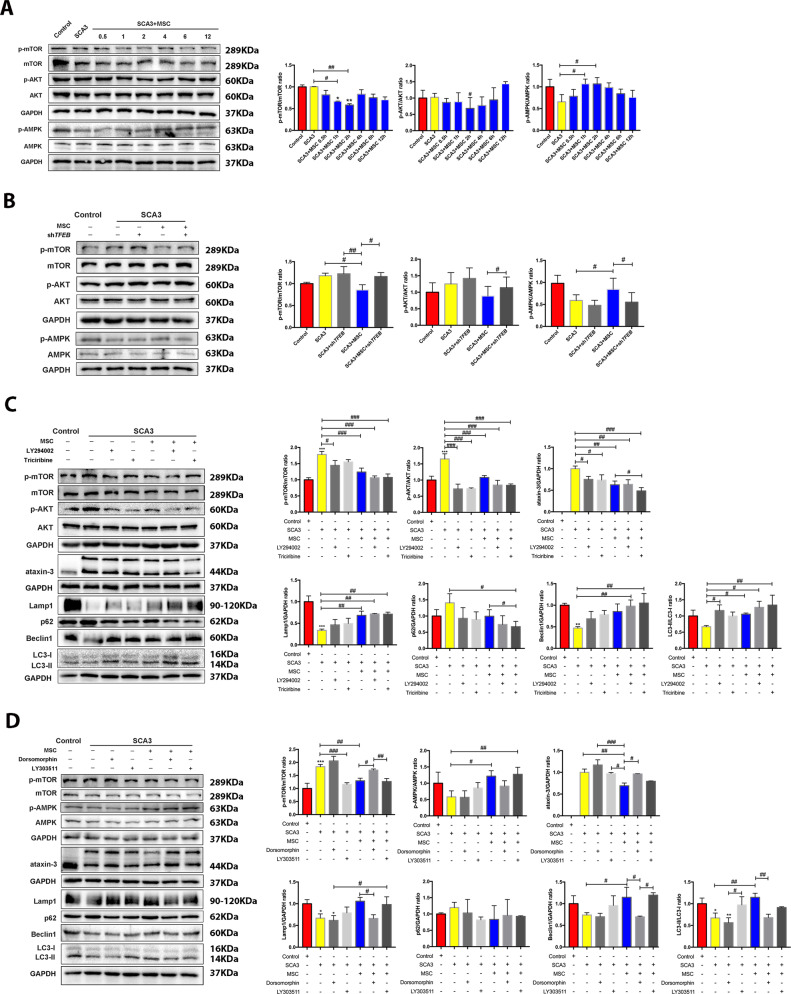
MSCs activated autophagy by regulating the AKT/mTOR and AMPK/mTOR signaling pathways to reduce the intracellular mutant protein ataxin-3. **A** The expression levels of p-AKT, p-mTOR, and p-AMPK at various times after MSCs therapy. **B** The effects of *shTFEB* on the expression of p-AKT, p-mTOR, and p-AMPK after MSCs. **C** The effect of LY294002 and triciribine on the expression of levels of p-AKT, p-mTOR, ataxin-3, and autophagy-related proteins after MSCs therapy. **D** The effect of LY303511 and dorsomorphin on the expression of levels of p-AKT, p-mTOR, ataxin-3, and autophagy-related proteins after MSCs therapy. *p < 0.05, **p < 0.01, ***p < 0.001 vs Control group, #*p* < 0.05, ^##^*p* < 0.01, ^###^*p* < 0.001.

Following mTORC1 regulated autophagy, and our mTORC1-TFEB findings prompted us to observe p-AKT and p-mTOR levels to evaluate MSCs co-culture effect on AKT/mTOR and AMPK/mTOR signaling pathways. As shown in Fig. 5A, MSCs co-culture treatment increased p-AMPK significantly within 2 h, suggesting that the treatment can inhibit AKT/mTOR and activate the phosphorylation of AMPK/mTOR signaling pathways in SCA3 patients compared to the *shTFEB*-treated SCA3 neurons (Fig. 5B).

To corroborate that the activity MSCs induces autophagy by regulating the levels of phosphorylation of AKT/mTOR and AMPK/mTOR signaling pathways, SCA3 neurons were treated with various phosphorylation inhibitors. Figure 5C, D showed that PI3K inhibitor LY294002 and AKT inhibitor Triviribine inhibited the phosphorylation of AKT and mTOR, further increasing the expression levels of LC3-II, Beclin1, and Lamp1, and decreasing the expression of p62 and mutant ataxin-3. Contrary, AMPK inhibitor Dorsomorphin inhibited AMPK phosphorylation and LC3-II, Beclin1, and Lamp1, and increased expression of p62 and mutant ataxin-3, while mTOR inhibitor LY303511 increased LC3-II, Beclin1, and Lamp1 and reduced the expression of p62 and ataxin-3. In addition, we also detected the expression of a downstream protein of the mTOR pathway. We realized that mTORC1 could activate the phosphorylation of S6. After MSCs treatment, p-mTOR decreased, resulting in decreased p-S6 (Fig. S3E). In general, these results showed a clear correlation between the activity of mTOR and the reduction of the intracellular mutant ataxin-3, indicating that SCA3 neurons autophagy is phosphorylation-dependent.

### TFEB-dependent MSCs therapy attenuated the toxic effects of mutant ataxin-3 in SCA3 neurons

**Fig. 6:**
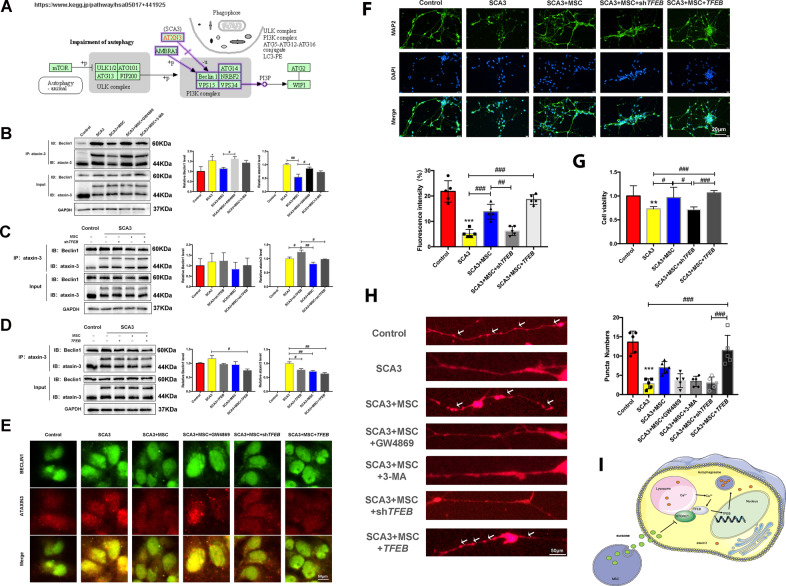
TFEB-dependent MSCs therapy attenuated Beclin1 ubiquitination of ataxin-3 protein and the toxic effects of mutant proteins in SCA3 neurons. **A** A diagram that comes from www.kegg.jp/pathway. **B** The effects of GW4869 and 3-MA on the interaction of Beclin1 and ataxin-3 using Co-IP. **C** The effects of *shTFEB* on the interaction of Beclin1 and ataxin-3 through Co-IP. **D** The effects of *TFEB* on the interaction of Beclin1 and ataxin-3 by Co-IP. **E** The effects of *shTFEB* and *TFEB* on the interaction of Beclin1 and ataxin-3 by immunofluorescence (scale bar: 50 μm). **F** The effects of *shTFEB* and *TFEB* on the neuron-specific marker MAP2 after MSCs by immunofluorescence (scale bar: 20 μm). **G** The effects of *shTFEB* and *TFEB* on neuron survival and proliferation. **H** The effects of *shTFEB*, *TFEB*, 3-MA, and GW4869 on the synaptic protein PSD95 after MSCs (scale bar: 50 μm). **I** Schematic diagram of the proposed mechanism. *p < 0.05, **p < 0.01, ***p < 0.001 vs Control group, ^#^*p* < 0.05, ^##^*p* < 0.01, ^###^*p* < 0.001.

To investigate the mechanism of MSCs therapy on the expression of mutated ataxin-3 protein through *TFEB* autophagy activation, we performed a Co-IP analysis. As shown in Fig. 6A, the mutant ataxin-3 protein competed with normal ataxin-3 for interaction with Beclin1, lowering intracellular Beclin1 levels, and consequently impairing intracellular autophagy reference. Next, we detected the interaction between ataxin-3 and Beclin1 protein after MSCs co-culture treatment. It was confirmed that MSCs reduced the intracellular level of mutant ataxin-3 by enhancing autophagy-lysosome function and alleviating the interaction between the mutant ataxin-3 and Beclin1, while autophagy inhibitor and the MSCs supernatant with GW4869 inhibited this effect (Fig. 6B). Subsequently, we examined the effect of *shTFEB* and overexpressed *TFEB* on the interaction between the two proteins. *shTFEB* enhanced the interaction between ataxin-3 and Beclin1 (Fig. 6C). However, overexpression of *TFEB* further enhanced the effect of MSCs and reduced the interaction between mutated ataxin-3 and Beclin1 (Fig. 6D). In addition, ataxin-3 and Beclin1 colocalized, which is consistent with the Co-PI assay (Fig. 6E). These results suggested that MSCs therapy reduced the expression of mutated ataxin-3 protein through *TFEB* activation of autophagy, thereby reducing the interaction between mutated ataxin-3 protein and Beclin1.

After verifying that MSCs affect autophagy flux and mutate ataxin-3 protein through the *TFEB* gene, we examined the effect of the *TFEB* gene on cell toxicity and survival proliferation. In MSCs-treated SCA3, overexpressed TFEB improved neuronal growth, while *shTFEB* inhibited the growth of axons and dendrites as well as the number of synapses on dendrites (Fig. 6F, H). Subsequently, the CCK8 assay also showed a significant increase in cell proliferation after MSCs co-culture, which was inhibited by *shTFEB*, while overexpression of TFEB further improved cell survival (Fig. 6G). Figure 6I shows the possible mechanism to summarize our whole study. These results showed that the effect of MSCs treatment depends on *TFEB* nuclear translocation.

## Discussion

SCA3 remains an incurable disease despite being known for decades. Efforts have been made to understand its mechanism and treatment. It is a polyQ disease characterized by CAG repeats that are closely related to the onset time and severity of the disease [33]. SCA3 patients possess more polyQ increases and ataxin-3 protein becomes poorly soluble, leading to a large number of inclusion bodies and eventually damaged neurons. Therefore, the most effective treatment is to inhibit or eliminate the mutant protein to improve the disease phenotype.

The balance between ubiquitin-proteasome and autophagy is essential for maintaining a balance between cell quality control and protein homeostasis. The ataxin-3 protein binds to the ubiquitinated protein through the UIM domain and acts as a ubiquitin hydrolase. So, ataxin-3 is a key factor binding in the ubiquitin-proteasome and autophagy pathway [1]. It has been reported that the wild-type ataxin-3 can interact with the autophagy protein, Beclin1, and that the deubiquitination activity of ataxin-3 protects Beclin1 from degradation by the proteasome [34]. However, mutated ataxin-3 competes with wild-type ataxin-3 to bind Beclin1, thereby destabilizing Beclin1 expression and autophagy process [35]. Consistent with our study, the expression of Beclin1, LC3 proteins in SCA3 patients were significantly reduced. After MSCs co-culture treatment, the levels of Beclin1, ULK1, and LC3 were significantly increased, the effect which was reversed with MSCs treatment and inhibited with autophagy inhibitor 3-MA attributable to the degradation of mutant ataxin-3 protein and reduction of mutated ataxin-3 and Beclin1 interaction. Consistent with this finding, Bafilomycin A1, an inhibitor of autophagy, significantly aggravated the neurotoxicity of ataxin-3 on retinal cells in the Drosophila model [36]. Moreover, Rapamycin has been shown to enhance autophagy and promote the degradation of ataxin-3 aggregation [37]. Besides, the overexpression of Beclin1 has been shown to promote autophagy and decrease cell death caused by ataxin-3 aggregates in the brain tissues of SCA3 patients and poly-ataxin-3 mice [38]. Other studies found that Beclin1 levels significantly reduced whereas ATG12, Lamp2, and LC3 significantly increased in the brain of SCA3 patients [39], corroborating our finding that autophagy increased in the early stages of the disease, p62 and LC3 located in ataxin-3 aggregates in the brain regions of SCA3 patients to clear mutant protein.

There is increasing evidence that cumulative enhancement of DNA damage and apoptosis were observed in SCA3 animal models and postmortem tissues of SCA3 patients [40, 41]; however, it is not clear what mediates ataxin-3 nuclear transfer to cause DNA damage [42]. It is known that the mutant polyQ protein accumulated in the nucleus to form intranuclear inclusion bodies, which can directly cause cell toxicity and DNA damage [43]. It has also been found that the mutant protein can interact with transcription factors in the nucleus to cause transcriptional dysregulation, such as TAFII130, Sp1, and p53 [44]. It can also interact with HMGB1/2 to inhibit the phosphorylation of kinase 1 and histone 2AX, thereby inhibiting the cellular response to the stress signal of the toxic response [45]. In addition, it was reported that the extended CAG repeat itself plays a toxic role in SCA3 pathogenesis [46]. Therefore, we examined the effect of MSCs on SCA3 cell DNA damage and apoptosis. MSCs co-culture therapy increased the survival rate of patients’ cells and decreased the expression of apoptotic proteins Bax and the release of lactate dehydrogenase, indicating that MSCs co-culture therapy enhanced autophagy flow and apoptosis inhibition. The interaction between Beclin1 and Bcl-2 is considered to be a potential regulator of autophagy and apoptosis [47]. MSCs co-culture therapy increased Beclin1 levels and dissociated from Bcl-2 to activate autophagy. Specifically, the role of Beclin1 and Bcl-2 in inducing autophagy and apoptosis by MSCs-mediated therapy needs to be further investigated [48, 49].

MSCs have great potential and advantages in the treatment of neurodegenerative diseases. They are easy to acquire and isolate without ethical issues from a variety of sources and have a strong paracrine effect because they can deliver neurotrophic factors, cytokines, exosomes, or mitochondria [50–52]. It is now widely believed that exosomes released by MSCs play an important role in cell treatment [53]. For instance, Jin et al., 2019 reported that exosomes secreted from adipose-derived stem cells attenuated diabetic nephropathy by promoting autophagy flux in podocytes [54]. In other studies, MSCs-mediated exosomes improved hepatic glucose and lipid metabolism in T2DM rats and rescued myocardial ischemia/reperfusion injury by activating autophagy [55, 56]. Moreover, ESC-exosomes treatment also alleviated hippocampal neural stem cells senescence and neuron differentiation capacity, and reversed cognitive impairment by inhibiting mTORC1 activation, thereby promoting TFEB nuclear translocation and lysosome resumption [57]. Therefore, we detected the existence of exosomes in MSC supernatant by WB, NTA, and TEM along with the exosome inhibitor GW4869 as a control. We found that the effect of MSCs co-culture therapy was inhibited by the addition of MSC supernatant without exosomes. The results showed that exosomes produced by MSCs could be inhibited by GW4869 [58]. Studies have compared the efficacy of a single intracranial injection with repeated systemic MSC administration in alleviating the MJD phenotype. They found that a single MSC transplant produced only a transient effect, whereas periodic administration promoted sustained motor behavior and neuropathological remission [59]. Therefore, we adopted the method of continuous co-culture therapy, adding new MSCs supernatant of medium change at the same time every day. The results also demonstrated that the intracellular mutation of ataxin-3 was reduced after 3 days of consecutive treatment.

Transcription Factor EB, a member of the MITF-TFE family, is known to be the most important regulator of the transcription of genes responsible for controlling lysosomal biogenesis and autophagy [60]. In vitro and in vivo studies have shown that *TFEB* plays an important role in metabolic diseases, immune diseases, cardiovascular diseases, cancer development, and neurodegenerative diseases [61–63]. Serine/threonine kinase mTOR plays a critical role in TFEB phosphorylation. mTOR has two complexes, mTORC1 and mTORC2. mTORC1 is mainly involved in cell growth, proliferation, survival, protein synthesis, and autophagy, while the mTORC2 complex is mainly involved in cell shape and metabolism, but it can also indirectly regulate autophagy [64, 65]. Under normal conditions, mTORC1 is recruited to the lysosome surface and promotes phosphorylation of TFEB. Phosphorylated TFEB remains inactivated in the cytoplasm. When mTORC1 is inactivated and separated from lysosomes under starvation or oxidative stress, TFEB dephosphorylation is transferred to the nucleus, and transcription of a CLEAR gene is induced, which activates lysosome and autophagy [66, 67]. Therefore, in our study, we examined the expression of TFEB and mTOR after MSCs co-culture therapy. Approximately 1 h after MSC treatment, TFEB decreased in the cytoplasm and increased in the nucleus. RT-qPCR results also showed that autophagy and lysosomal-related genes were significantly elevated, while phosphorylation of mTOR decreased. These results suggest that mTOR inactivation leads to nuclear translocation of dephosphorylated TFEB after MSCs treatment, which activates lysosome and autophagy. Meanwhile, we also detected the expression of the intracellular mutant protein ataxin-3 by interference and overexpression of TFEB. After MSCs treatment, the mutant protein ataxin-3 was significantly reduced. The reduced mutant protein ataxin-3 was reversed after interfering with TFEB by lentivirus. Overexpression of TFEB further reduced ataxin-3 expression and improved neuronal growth. This is consistent with several studies of TFEB in the elimination of accumulated mutant proteins in neurodegenerative diseases [20, 68–70]. However, studies found that excessive induction of TFEB is linked with carcinogenic risk, suggesting that TFEB may regulate tumor genesis. Therefore, TFEB needs to be activated intermittently and/or for a limited time to avoid potential carcinogenic effects [71, 72].

mTORC1 is a major regulator of autophagy [73, 74]. It’s important to understand how mTORC1 regulates autophagy since it can link cellular signaling pathways to autophagy regulation [75]. The PI3K/AKT pathway is a major modulator upstream of mTORC1 and is common in neurodegenerative diseases such as Alzheimer’s disease and Parkinson’s disease [76]. AMPK also regulates autophagy through the negative regulation of mTORC1, which participates in autophagy initiation [77, 78]. AMPK also supports nuclear translocation and activation of TFEB with the mTORC1 pathway [79, 80]. Therefore, in our study, we detected the expression of the PI3K/AKT/AMPK/mTORC1 pathway after MSCs treatment. The levels of p-AKT and p-mTOR decreased, while the levels of p-AMPK increased significantly. The phosphorylation changes were reversed with the addition of PI3K, AKT, and AMPK inhibitors. However, the expression levels of LC3-II and Beclin1 further increased after adding the mTOR inhibitors. Collectively, these data suggest that MSCs co-culture therapy-induced autophagy through the PI3K/AKT /AMPK/mTOR pathway.

In summary, our research demonstrated that MSCs co-culture therapy attenuated intracellular mutated ataxin-3 proteins by activating TFEB nuclear translocation-dependent autophagy. Our results provide a new therapeutic target for SCA3 disease and other neurodegenerative diseases through MSCs therapy.

## Materials and methods

### Reagents and antibodies

The supernatant was obtained from the MSC medium with or without GW4869 that were cultured for 3 days. The MSC medium with or without GW4869 that were cultured for 3 days was ultracentrifuged to get exosomes. The exosomes were collected and resuspended in sterile 1×PBS for future use.

LY294002 (20 μM), LY303511 (10 μM), Triciribine (10 μM), mTOR, HSP70, CD9, CD63, and ULK1 were purchased from Beyotime (Shanghai, China). 3-MA (10 mM) was purchased from Selleckchem (Houston, USA). GW4869 (10 μM) was purchased from Targetmol (Boston, USA). Chloroquine (20 μM) was purchased from Merck (New Jersey, USA). Dorsomorphin (10 μM) was purchased from Glpbio (California, USA). p-AMPK/AMPK, p-mTOR(ser2448) were purchased from Wanleibio (Shenyang, China). p-AKT/AKT1/2/3 were purchased from Abmart (Shanghai, China). Bax, Bcl-2, Caspase3, Lamp1, LC3B, Beclin1, p-S6 (ser235/236), and p62 were purchased from Cell Signaling Technology (Boston, USA). Ataxin-3 was purchased from Millipore (Massachusetts, USA). GAPDH and p-TFEB (ser142) was purchased from Bioss (Beijing, China). TFEB was purchased from Abcam (Massachusetts, USA). Detailed information is listed in the Supplementary Table.

### Western blotting analysis and co-immunoprecipitation

All the cells in the culture dish were lysed in RIPA buffer (Beyotime, Shanghai, China) containing PMSF solution (GenStar, Beijing, China) on ice. The nuclear and cytosolic proteins were obtained using a nuclear/cytosol fractionation kit (EpiZyme, Shanghai, China). The agarose was obtained from protein A + G (Beyotime, Shanghai, China). The lysis buffer was used for both Western blotting and co-immunoprecipitation experiments. After denaturation, protein samples were separated on a PAGE Gel Fast Preparation Kit (EpiZyme, Shanghai, China) and transferred onto PVDF membranes (Millipore, Massachusetts, USA). After incubating with primary antibodies at 4 °C overnight and HRP-conjugated secondary antibodies (Beyotime, Shanghai, China) at room temperature for 2 h. Chemiluminescence Kit (Beyotime, Shanghai, China) was used for protein bands visualization.

### Cell culture and differentiation of iPSC-derived neurons

The iPSCs were obtained by reprogramming the urinary cells from SCA3 patients (Ethics number: (No. 201412458) as described in the previous work [81]. Then, iPSCs were cultured on 5 μL/mL Matrigel (Corning, New York, USA) with mTeSR™1 (Stemcell Technologies, Vancouver, Canada). After 3 days in mTeSR™1 medium, iPSCs were re-plated onto Matrigel-coated 12-well plates in N2B27 with 2i inhibitor medium (1:1 mixture of N2 and B27). The N2 medium consisted of 1×N2, DMEM/F-12, NEAA (1×), GlutaMAX (1×), 5 μg/mL insulin, 1 mM l-glutamine, 100 μM 2-mercaptoethanol. The B27 medium contained Neurobasal, 1 × B27, 5 μM SB431542, and 5 μM dorsomorphin). After 7 days in N2B27 + 2i medium, iPSCs were re-plated onto Matrigel-coated 6-well plates in N2B27 medium to obtain visible neural rosettes, which were picked and dissociated into single cells in accutase (Sigma, Missouri, USA). Next, single cells were re-plated onto Matrigel-coated 24-well plates in an N2B27 medium for more than 1 month to obtain neurons.

Mesenchymal stem cells derived from cord blood were cultured with DMEM/F-12 + 10% FBS. After 2 days of passaging, the cells were added with exosome inhibitors GW4869 + DMEM/F-12 (without FBS). On the third day, the cell supernatant was collected and centrifuged at low speed to remove cell debris and dead cells. We mixed the cell supernatant with N2B27 medium in the same proportion and added the mixture to SCA3 neural cells. The mixture was repeatedly changed on a daily basis for three consecutive days.

### CCK8 assay

Cell viability was determined by the CCK8 assay (Beyotime, Shanghai, China). According to the manufacturer’s instructions, cells in 96-well plates were added with CCK8 solution and incubated for 0.5–1 h at 37 °C. Then, the absorbance of each well was measured at 450 nm.

### Lactate dehydrogenase (LDH) cytotoxicity assay

Cell apoptosis was measured by the LDH Cytotoxicity Assay Kit (Beyotime, Shanghai, China). Briefly, 1 × LDH solution (diluted with PBS) was added to the cells in 96-well plates and incubated in the dark for 0.5 h at room temperature. Then, the absorbance value was measured with a microplate reader (Berthold, LB943, Germany) at 490 nm.

### Immunofluorescence

The cells in the culture dish were fixed in 4% paraformaldehyde for 15 min, then permeabilized with 0.5% Triton X-100 for 5 min, and blocked with 10% bovine serum albumin (BSA). Next, the cells were incubated with specific primary antibodies overnight at 4 °C and then incubated with secondary antibodies in 10% BSA. Nuclei were counterstained with DAPI for 5 min. Images were acquired with a fluorescence microscope (Olympus, IX73, Japan). The primary anti-stem cell markers were Map2 (Millipore), NANOG (Cell Signaling Technology), Nestin, Pax6, SSEA4 (Abcam, Massachusetts, USA), and PSD95 (Invitrogen, Massachusetts, USA).

### Quantitative real-time PCR

Total RNAs were extracted using Trizol reagent (Invitrogen, Massachusetts, USA) and reverse-transcribed using the HiScript II Q RT SuperMix (Vazyme, Nanjing, China). Gene expression values were normalized against that of GAPDH. Then, qRT-PCR was performed by using specific primers in a CFX96 Real-Time System (Bio-Rad, USA). Primer sequences for qRT-PCR were obtained from IGEbio (Guangzhou, China) and are listed in Supplementary Table.

### Flow cytometry

The cells were fixed in 4% paraformaldehyde for 30 min at 37 °C. Then, the cells were resuspended in PBS buffer and incubated with the primary antibodies overnight at 4 °C and incubated with secondary antibodies for 30 min at room temperature. After washing with PBS, the cells were resuspended in 200–300 μL of PBS and proceeded for analysis on BD Accuri C6 Plus (New Jersey, USA).

### Acridine orange staining

According to the manufacturer’s instructions, the cells in 12-well plates were added with AO solution (Real-times, Beijing, China) and incubated in the dark for 0.25–0.5 h at 37 °C. After washing three times with PBS, the cells were observed with a fluorescence microscope at 488 nm.

### Lyso-tracker red assay

The Lysosomes in living cells were determined by the Lyso-Tracker Red Assay Kit (Beyotime, Shanghai, China). Before this experiment, Lyso-Tracker Red working fluid was preheated at 37 °C. The cells in 12-well plates were added with Lyso-Tracker Red working solution and incubated for 0.1–1 h at 37 °C. Then, the medium was changed to a fresh medium and observed with a fluorescence microscope at 577 nm.

### Fluo-4 AM assay

For detecting intracellular Ca^2+^, Fluo-4 AM Assay Kit (Beyotime, Shanghai, China) was used. After washing three times with PBS, the cells in 96-well plates were added with Fluo-4 AM solution (Diluted with PBS) and incubated for 0.5–1 h at 37 °C. Then, the cells were washed three times with PBS again, and the absorbance of each well was measured at 488 nm.

### Cell transfection

The Lentiviral Plasmid (pLKO.1-U6-homo-sh*TFEB*-EF1a-copGFP-T2A-puro, pCDH-CMV-Homo-*TFEB*-EF1-copGFP-T2A-Puro) was obtained from IGEbio (Guangzhou, China). After co-transfection of lentiviral plasmid (psPAX2 and pMD2.G) into 293 T cells, the medium was collected to infect neuron cells. The neuron cells in six-well plates were added with lentiviral supernatant and polybrene (Invitrogen, Massachusetts, USA) and incubated for 24 h at 37 °C. After 24 h of transfection, the medium was changed to N2B27 medium and continuously cultivated for 48 h. Then the neuron cells were detected by immunofluorescence and western blotting.

### Transmission electron microscope

A transmission electron microscope (TEM) was used to observe the morphology of exosomes. After the extraction of exosomes by ultracentrifugation and fixation with 2.5% glutaraldehyde, the specimens were observed under the transmission electron microscope (Tecnai G2 Spirit, USA).

### Teratoma formation experiment

Teratoma formation experiment was used to demonstrate pluripotency of iPSCs, as it exhibited its ability to differentiate into tissues of all three germ layers (ectoderm, mesoderm, endoderm) in vivo. The iPSCs were injected into the axillary skin and the muscles of the hind legs of the mouse, and tumors were formed in about 2 months. Then the tumors were taken for HE staining.

### Nanoparticle tracking analysis

Nanoparticle tracking analysis (NTA) technology was used to determine the concentration and particle size of exosomes. After the exosomes were extracted by ultracentrifugation, they were resuspended in PBS and measured under the NTA.

### Statistical analysis

All the experimental data were performed using GraphPad Prism 7 with a one-way ANOVA for multiple groups comparison and a *t*-test for two groups comparison. All data were presented as mean ± SD at least three independent experiments. Statistical significance was defined as a *P* value <0.05.

## Supplementary information

supplementary materials

supplementary Figure 1

supplementary Figure 2

supplementary Figure 3

checklist

Original Data File

## Data Availability

The data that support the findings of this study are available from the corresponding author upon reasonable request.
