# Correction: Transcription factor EB-mediated mesenchymal stem cell therapy induces autophagy and alleviates spinocerebellar ataxia type 3 defects in neuronal cells model

**DOI:** 10.1038/s41419-022-05119-7

**Published:** 2022-07-28

**Authors:** Xiaobo Han, Jean de Dieu Habimana, Amy L. Li, Rongqi Huang, Omar Mukama, Weiyue Deng, Ling Wang, Yuying Zhang, Wei Wang, Sihao Deng, Kexin Peng, Bin Ni, Shusheng Zhang, Jufang Huang, Xiao-xin Yan, Zhiyuan Li

**Affiliations:** 1 CAS Key Laboratory of Regenerative Biology, Guangdong Provincial Key Laboratory of Stem Cell and Regenerative Medicine, Guangzhou Institutes of Biomedicine and Health, Chinese Academy of Sciences, Guangzhou, China; 2 Department of Anatomy and Neurobiology, Xiangya School of Medicine, Central South University, Changsha, China; 3 NHC Key Laboratory of Birth Defect for Research and Prevention, Hunan Provincial Maternal and Child Health Care Hospital, Changsha, China; 4 Changsha Stomatological Hospital, Changsha, China; 5 Bioland Laboratory, Guangzhou, China; 6 GZMU-GIBH Joint School of Life Sciences, Guangzhou Medical University, Guangzhou, China; 7 GIBH-HKU Guangdong-Hong Kong Stem Cell and Regenerative Medicine Research Centre, GIBH-CUHK Joint Research Laboratory on Stem Cell and Regenerative Medicine, Guangzhou, China

**Keywords:** Spinocerebellar ataxia, Neurodegeneration

Correction to: *Cell Death & Disease* 10.1038/s41419-022-05085-0, published online 18 July 2022

The original version of this article contained a mistake in an author name. Bing Ni needs to be corrected to Bin Ni. In addition, the affiliations of the corresponding author should be 1, 2, 3, 4, 5, 6, 7. The original article has been corrected.

