# Supplementary material for: Transcription factor EB-mediated mesenchymal stem cell therapy induces autophagy and alleviates spinocerebellar ataxia type 3 defects in neuronal cells model
Source: Cell Death Dis. 2022 Jul 18;13(7):622. doi: 10.1038/s41419-022-05085-0 (PMC9293975; doi:10.1038/s41419-022-05085-0)
Supplement: Supplementary file 1 — supplementary materials [file 41419_2022_5085_MOESM1_ESM.docx]

**Supplementary Fig. 1 The establishment of iPSCs-derived neuron cell models in SCA3 patients.**  **A** Karyotype analysis of SCA3 iPSCs. **B** iPSCs related markers were identified by flow cytometry analysis. **C** iPSCs related markers were identified by RT-qPCR. **D** Immunohistochemistry of differentiated iPSCs into 3 different layers (scale bar: 100 μm). **E** NCs related marker Beta Tubulin was identified by immunofluorescence (scale bar: 50 μm).

**Supplementary Fig. 2 MSCs therapy alleviated the toxic effects of mutant proteins on neurons.** **A** The survival and proliferation were measured by CCK-8 assay. **B** The levels of lactate dehydrogenase in the culture medium were measured by LDH assay. **C** The expression of apoptosis-related proteins Bcl-2, Bax, and Caspase3/cleaved-Caspase3 were identified by Western blotting after MSCs therapy. **D** Neurons specific marker MAP2 was measured by immunofluorescence (scale bar: 50 μm). * *p* < 0.05, ** *p* < 0.01, *** *p* < 0.001 vs Control group, ^#^ *p* < 0.05, ^##^*p* < 0.01,^###^*p* < 0.001.

**Supplementary Fig. 3 The effects of *shTFEB* and *TFEB* on the expression of p-mTOR and lysosome in SCA3 neuron. A** The expression of TFEB after adding Lentiviral *shTFEB* plasmid (scale bar: 50 μm). **B** The expression of TFEB after adding Lentiviral *TFEB* plasmid (scale bar: 20 μm). **C** The effects of *shTFEB* on the expression of p-mTOR/mTOR after MSCs therapy. **D** The effects of *shTFEB* and *TFEB* on the expression of lysosom by lysosome probe (scale bar: 50 μm). **E** The expression of downstream protein p-S6 of mTOR was identified by Western blotting after MSCs therapy.

**Supplementary Table**

Primer sequences for RT-qPCR analyses

| **Gene** | **Sequence (5'->3')** |
| --- | --- |
| *Atg5* | AAAGATGTGCTTCGAGATGTGT (F) |
|  | CACTTTGTCAGTTACCAACGTCA (R) |
| *Atg7* | ATGATCCCTGTAACTTAGCCCA (F) |
|  | CACGGAAGCAAACAACTTCAAC (R) |
| *Atg10* | CCCTTGGATGATTGTGAAGTGA (F) |
|  | CTGTAGCAGTCGCATCTTATAGC (R) |
| *Atg12* | CTGCTGGCGACACCAAGAAA (F) |
|  | CGTGTTCGCTCTACTGCCC (R) |
| *SQSTM1/p62* | GACTACGACTTGTGTAGCGTC (F) |
|  | AGTGTCCGTGTTTCACCTTCC (R) |
| *Lc3b* | AGCTCATCAAGATAATTAGAAGGCG (F) |
|  | AAACAATTCTAGAAGAGCTGCATT (R) |
| *Beclin1* | ACCTCAGCCGAAGACTGAAG (F) |
|  | AACAGCGTTTGTAGTTCTGACA (R) |
| *Tfeb* | TCCAACAAGGGAAGGTGACAT (F) |
|  | GCGCAACCCTATGCGTGA (R) |
| *Lamp2A* | GAAAATGCCACTTGCCTTTATGC (F) |
|  | AGGAAAAGCCAGGTCCGAAC (R) |
| *Lamp1* | TCTCAGTGAACTACGACACCA (F) |
|  | AGTGTATGTCCTCTTCCAAAAGC (R) |
| *Ctsb* | AGAGTTATGTTTACCGAGGACCT (F) |
|  | GATGCAGATCCGGTCAGAGA (R) |
| *Ctsd* | ATTCAGGGCGAGTACATGATCC (F) |
|  | CGACACCTTGAGCGTGTAG (R) |
| *Gns* | GCATGACACCGCTAAAGAAAAC (F) |
|  | CACAACGTGATGATTATGTGGGT (R) |
| *Gba* | ATGGAGCGGTGAATGGGAAG (F) |
|  | GTGCTCAGCATAGGCATCCAG (R) |
| *Gapdh* | АСАСССАСТССТССАССТТТ (F) |
|  | TTACTCCTTGGAGGCCATGT (R) |
| *Nanog* | TGAACCTCAGCTACAAACAG (F) |
|  | TGGTGGTAGGAAGAGTAAAG (R) |
| *Oct4* | CCTCACTTCACTGCACTGTA |
|  | CAGGTTTTCTTTCCCTAGCT (R) |
| *Sox2* | CCCAGCAGACTTCACATGT (F) |
|  | CCTCCCATTTCCCTCGTTTT (R) |
| *Pax6* | TTGCTTGGGAAATCCGAG (F) |
|  | TGCCCGTTCAACATCCTT (R) |
| *Nestin* | CCACCCTGCAAAGGGAATCT (F) |
|  | GGTGAGCTTGGGCACAAAAG (R) |
| *Foxg1* | AGGAGGGCGAGAAGAAGAAC (F) |
|  | TCACGAAGCACTTGTTGAGG (R) |
| *Sox1* | TACAGCCCCATCTCCAACTC (F) |
|  | GCTCCGACTTCACCAGAGAG (R) |

(F) and (R) stand for forward and reverse primer, respectively.

﻿

Experimental Antibody

| **Name** | **Brand + Number** | **Concentration** |
| --- | --- | --- |
| **Antibody** | | |
| Chicken anti-Nestin | Abcam+ab134017 | 1:10000 |
| Rabbit anti-PAX6 | Abcam+ab5790 | 1:50 |
| Rabbit anti-MAP2 | Millipore+ab5622 | 1:300 |
| Anti-Beta III Tubulin-488 | Millipore+ab15708A4 |  |
| Rabbit anti-SSEA4 | Bioss+bs309R | 1:200 |
| Rabbit anti-ULK1 | Beyotime+AF8307 | 1:1000 |
| Rabbit anti-p-TFEB | Bioss+bs22337R | 1:1000 |
| Rabbit anti-SOX2 | Cell Signaling+D3579 | 1:400 |
| Mouse anti-ataxin-3 | Millipore+MAB5360 | 1:1000 |
| Rabbit anti-LC3B | Cell Signaling+3868T | 1:1000 |
| Rabbit anti-Beclin1 | Cell Signaling+3495T | 1:1000 |
| Rabbit anti-p62 | Cell Signaling+8025T | 1:1000 |
| Rabbit anti-TFEB | Abcam+ab270604 | 1:1000 |
| Rabbit anti-Lamp1 | Cell Signaling+9091 | 1:1000 |
| Rabbit anti-Bax | Cell Signaling+2722 | 1:1000 |
| Rabbit anti-Bcl-2 | Cell Signaling+15071 | 1:1000 |
| Rabbit anti-Caspase-3 | Cell Signaling+9662s | 1:1000 |
| Rabbit anti-p-S6 | Cell Signaling+4858 | 1:2000 |
| Rabbit anti-AKT | Abmart+T55561 | 1:1000 |
| Rabbit anti-p-AKT | Abmart+T40067 | 1:1000 |
| Rabbit anti-AMPK | Wanleibio+WL02254 | 1:1000 |
| Rabbit anti-p-AMPK | Wanleibio+WL05103 | 1:1000 |
| Rabbit anti-mTOR | Beyotime+AF1648 | 1:1000 |
| Rabbit anti-p-mTOR | Wanleibio+WL03694 | 1:1000 |
| Rabbit anti-GAPDH | Bioss+bs0755R | 1:1000 |
| Rabbit anti-Histone H3 | Abcam+ab1791 | 1:1000 |
| Rabbit anti-HSP70 | Beyotime+AF0189 | 1:1000 |
| Rabbit anti-CD9 | Beyotime+AF0108 | 1:1000 |
| Rabbit anti-CD63 | Beyotime+AF1471 | 1:1000 |
| **Secondary Antibodies** |  |  |
| Anti-Rabbit-488 | Proteintech+SA00006–2 | 1:400 |
| Anti-Mouse-594 | Proteintech+SA00006–3 | 1:400 |
| Anti-Chicken-594 | Jackson 703-585-155 | 1:400 |
| Anti-Mouse-488 | Invitrogen A32766 | 1:400 |
| Anti-Rabbit-594 | Invitrogen R37119 | 1:400 |
| HRP Goat anti-Mouse lgG | Beyotime+A0216 | 1:1000 |
| HRP Goat anti-Rabbit lgG | Beyotime+A0208 | 1:1000 |
