# Supplementary figures and images for: Transcription factor EB-mediated mesenchymal stem cell therapy induces autophagy and alleviates spinocerebellar ataxia type 3 defects in neuronal cells model
Source: Cell Death Dis. 2022 Jul 18;13(7):622. doi: 10.1038/s41419-022-05085-0 (PMC9293975; doi:10.1038/s41419-022-05085-0)

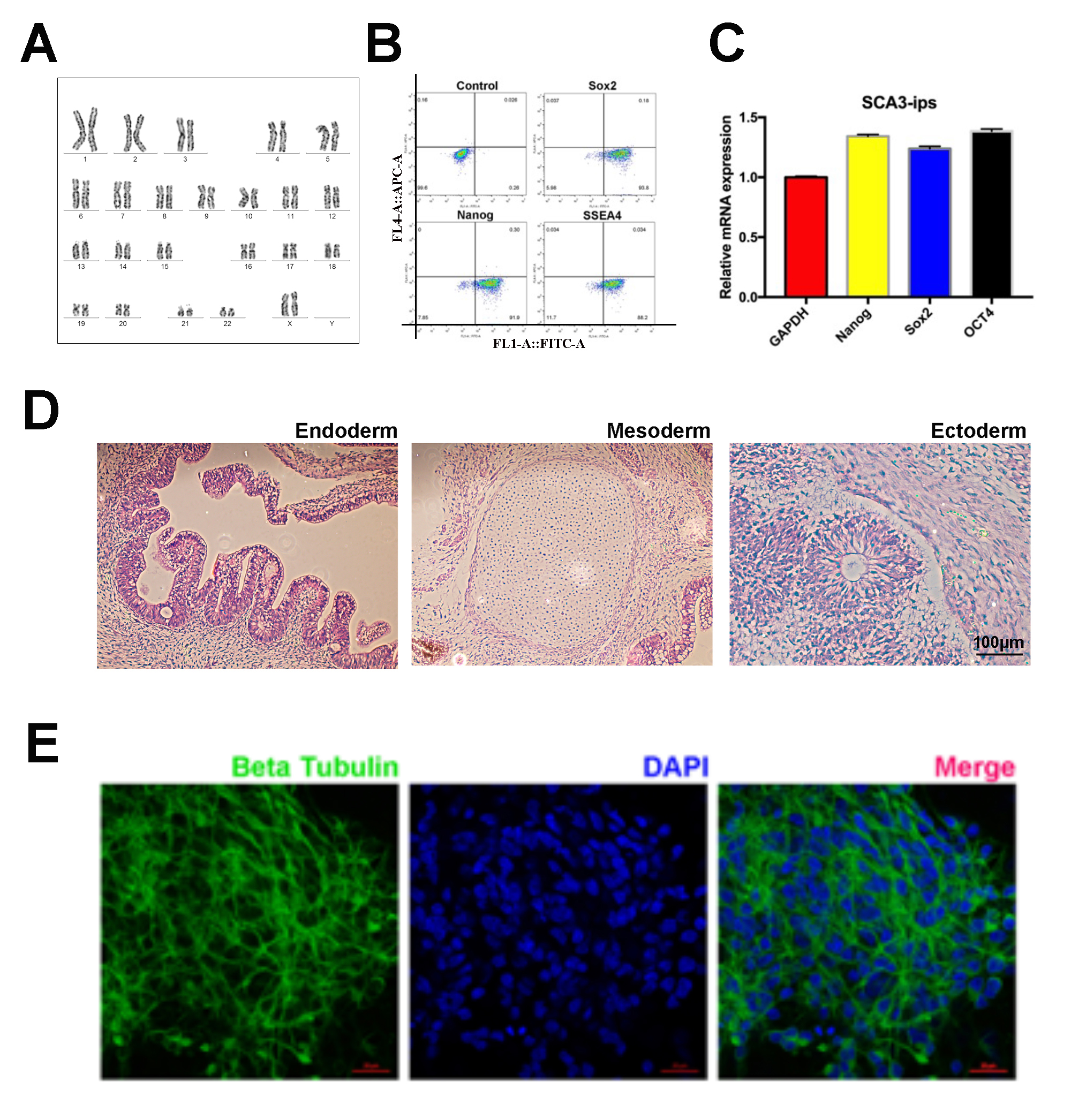

Supplement: Supplementary file 2 — supplementary Figure 1 [file 41419_2022_5085_MOESM2_ESM.jpg]

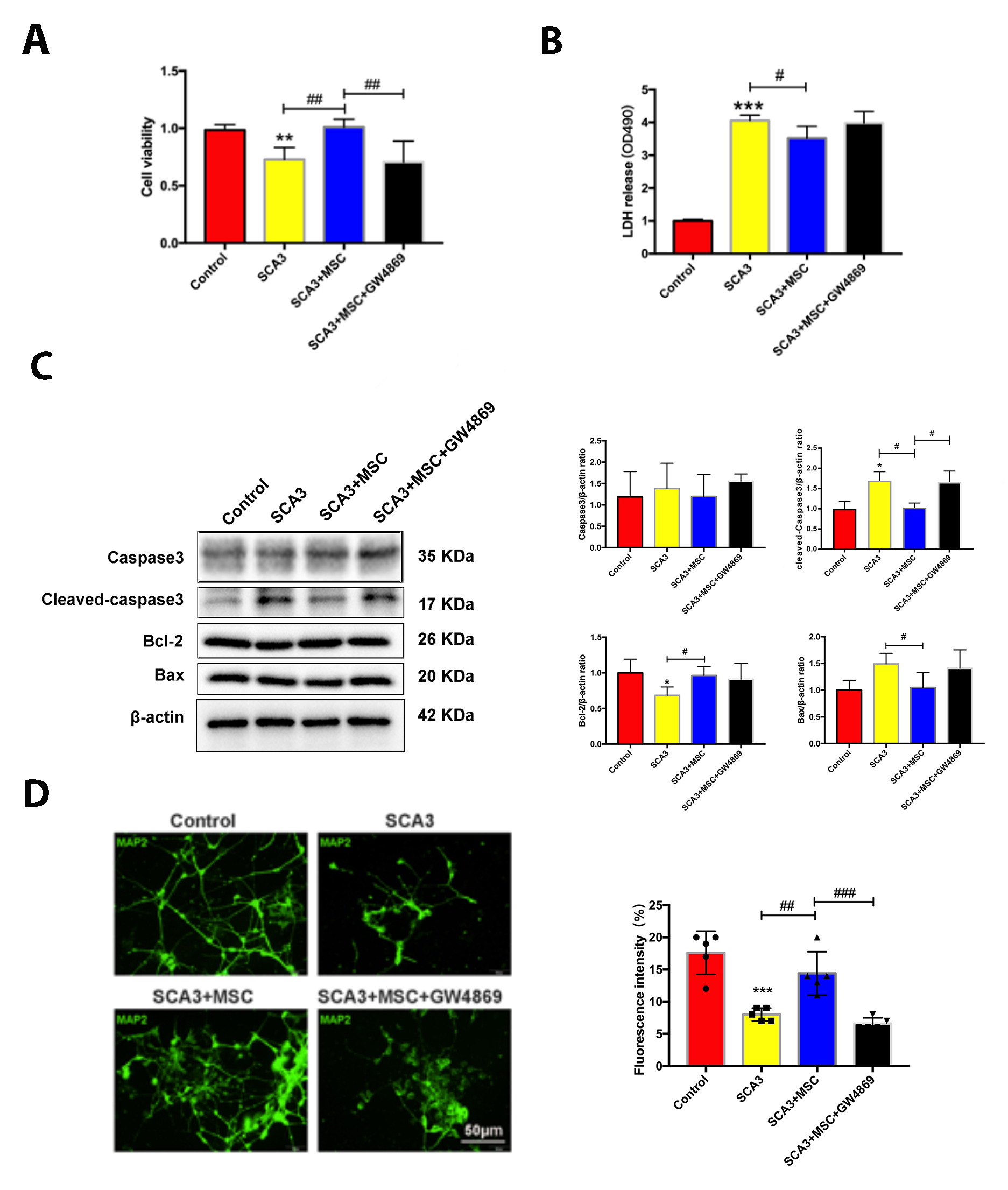

Supplement: Supplementary file 3 — supplementary Figure 2 [file 41419_2022_5085_MOESM3_ESM.jpg]

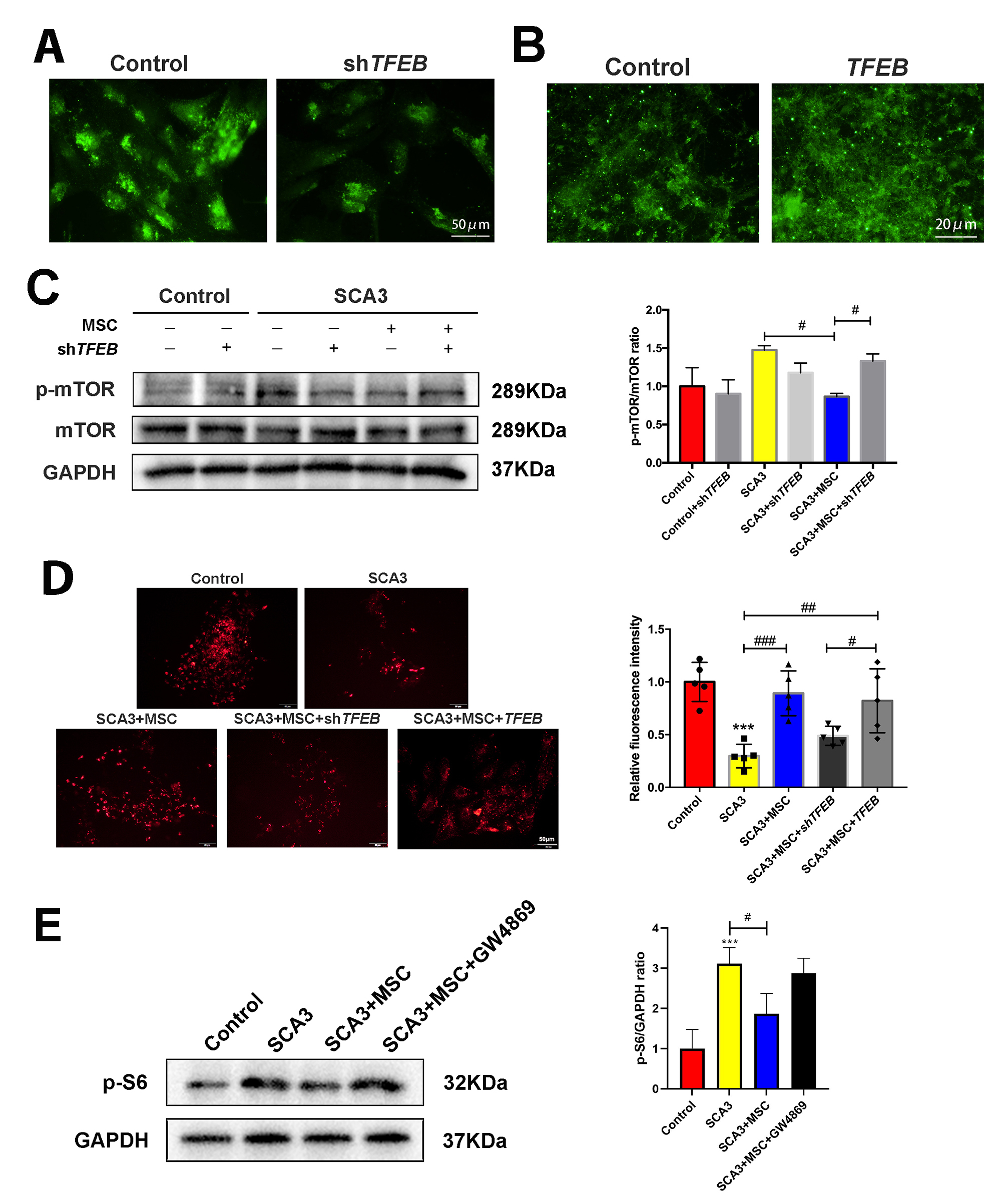

Supplement: Supplementary file 4 — supplementary Figure 3 [file 41419_2022_5085_MOESM4_ESM.jpg]

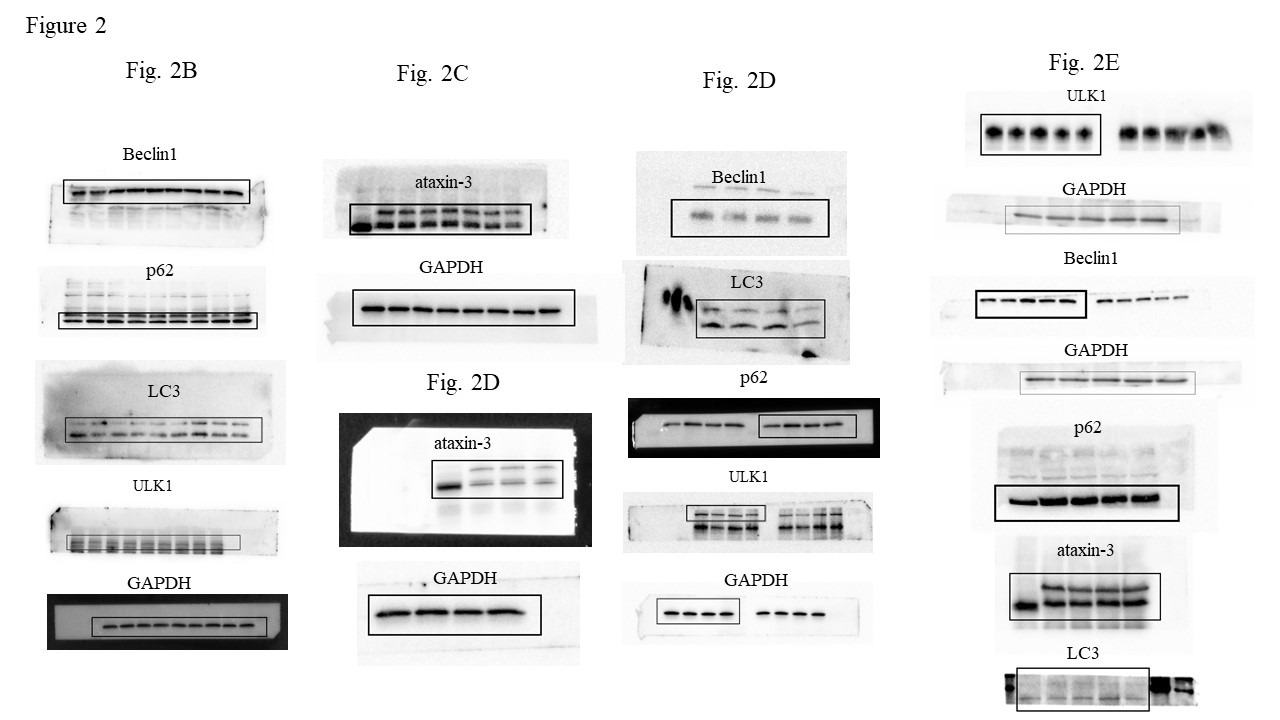

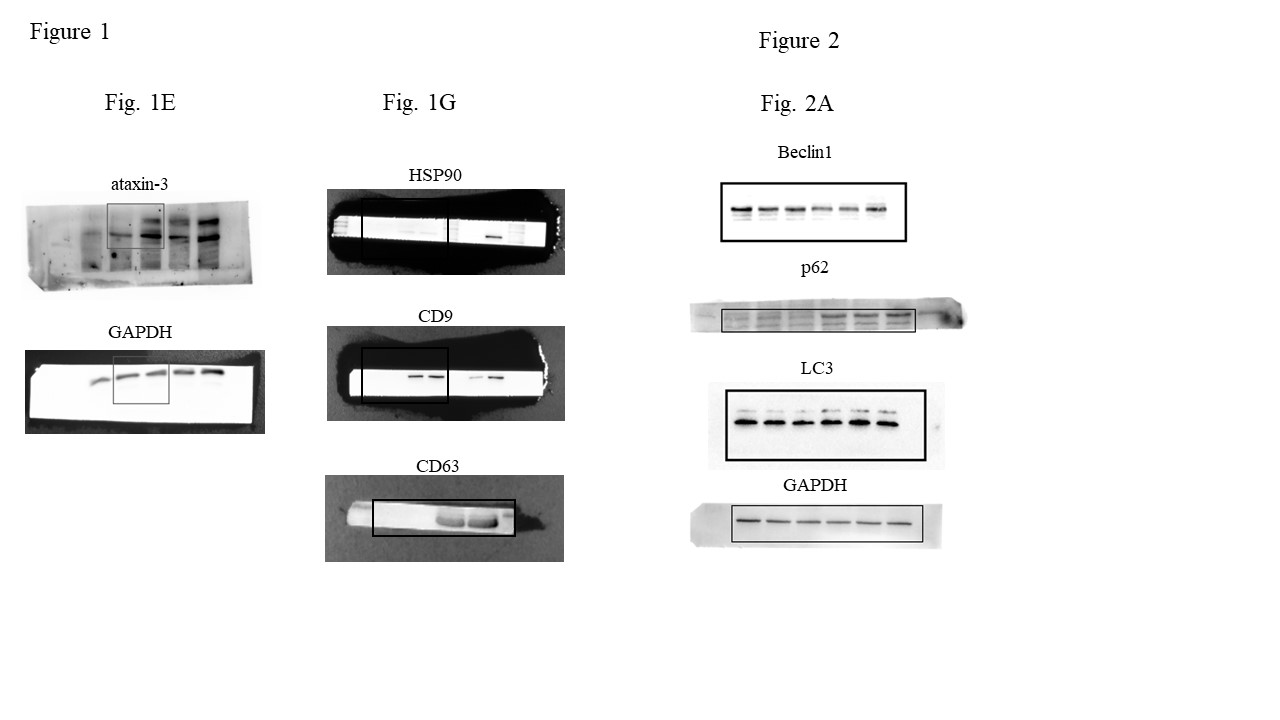


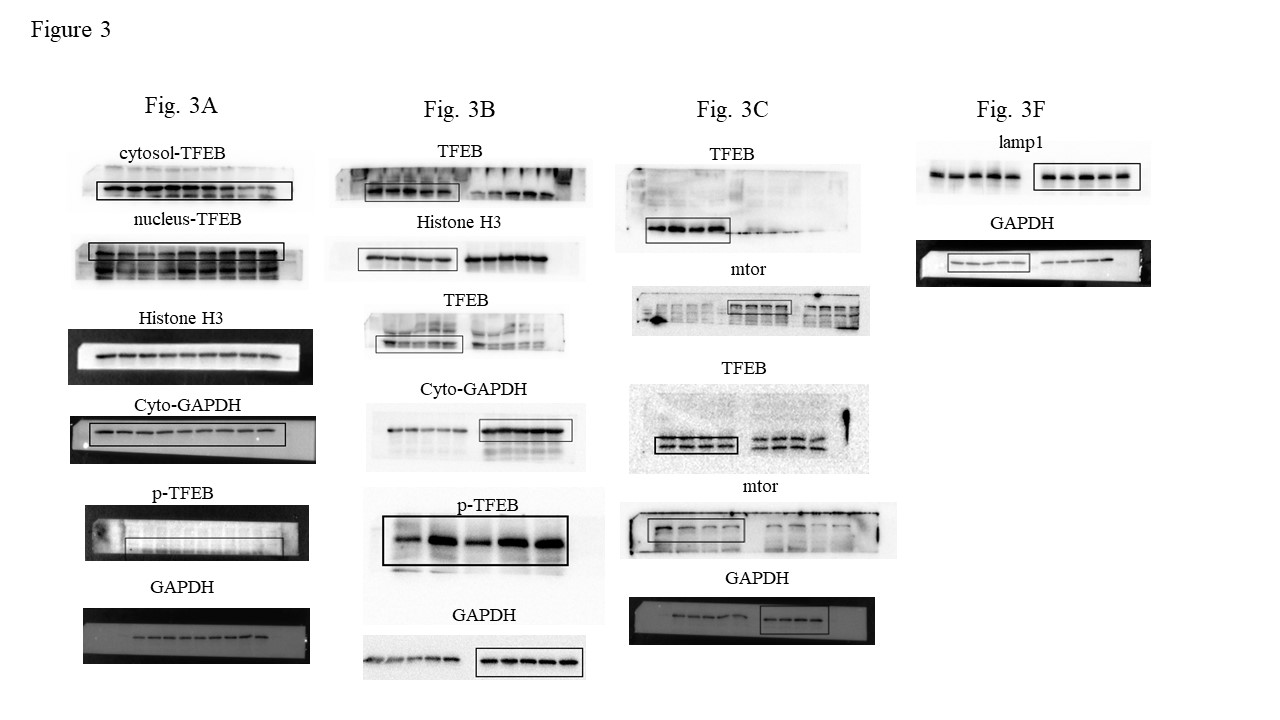


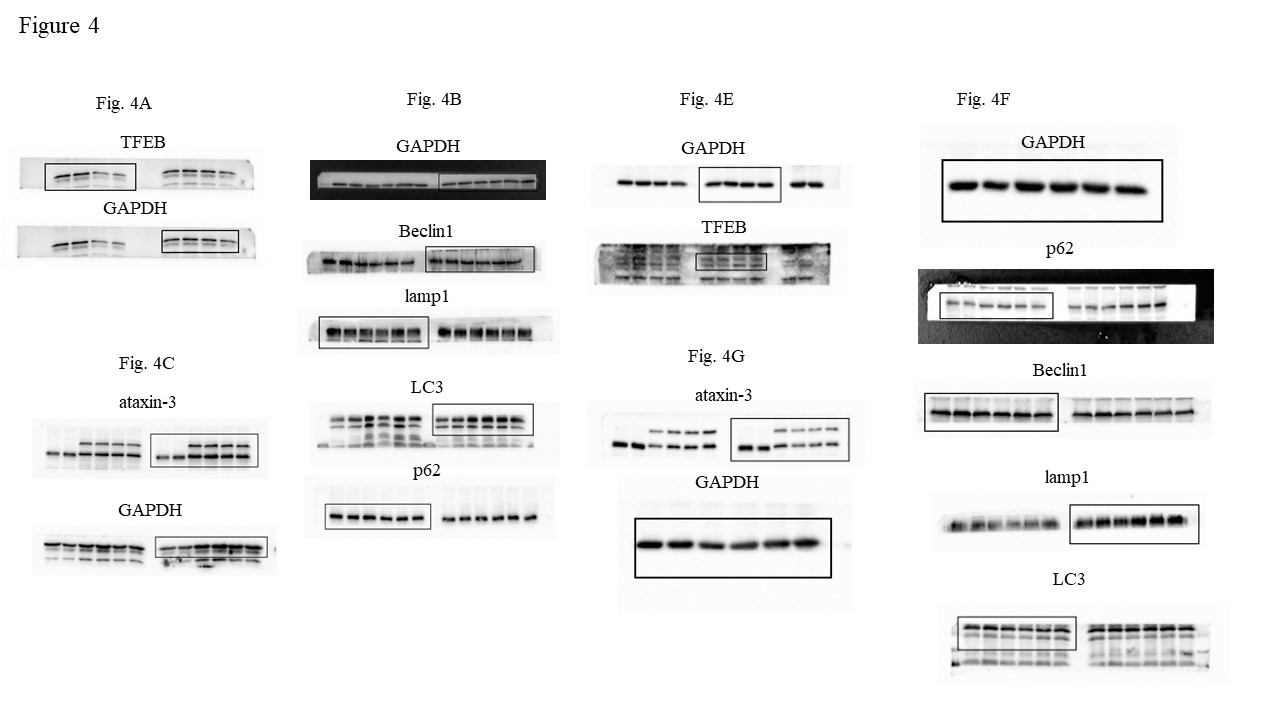


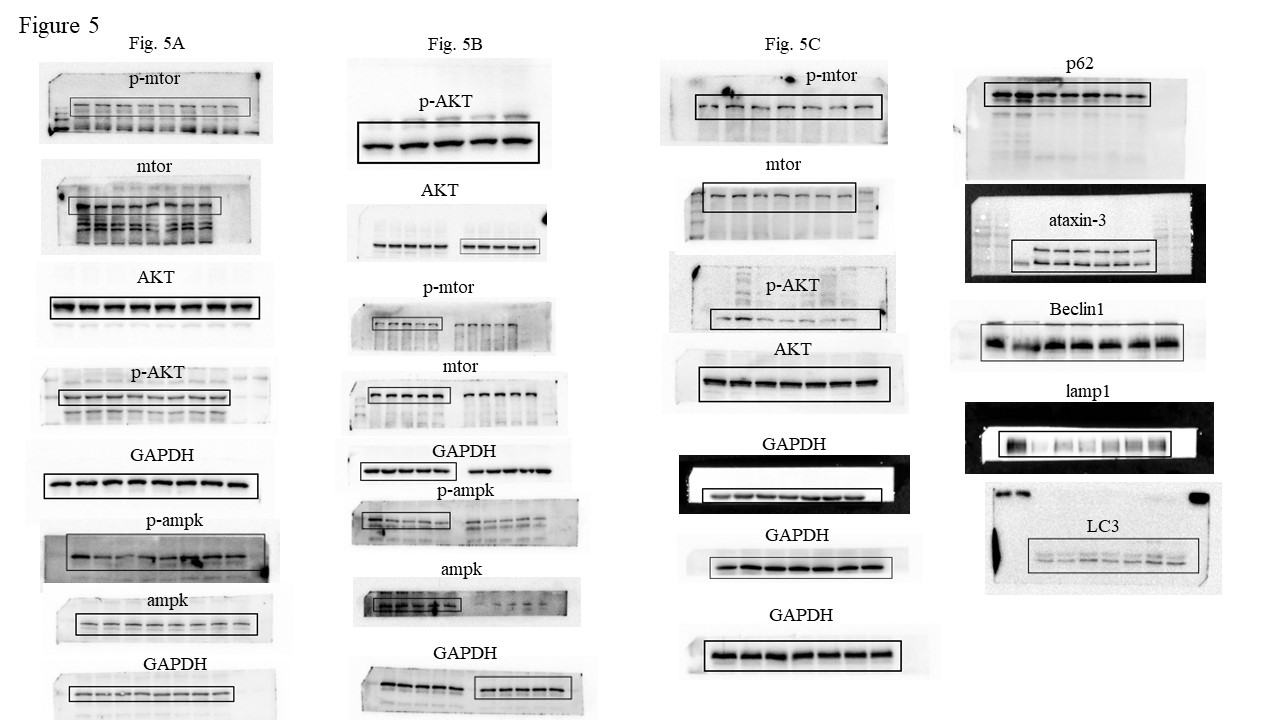


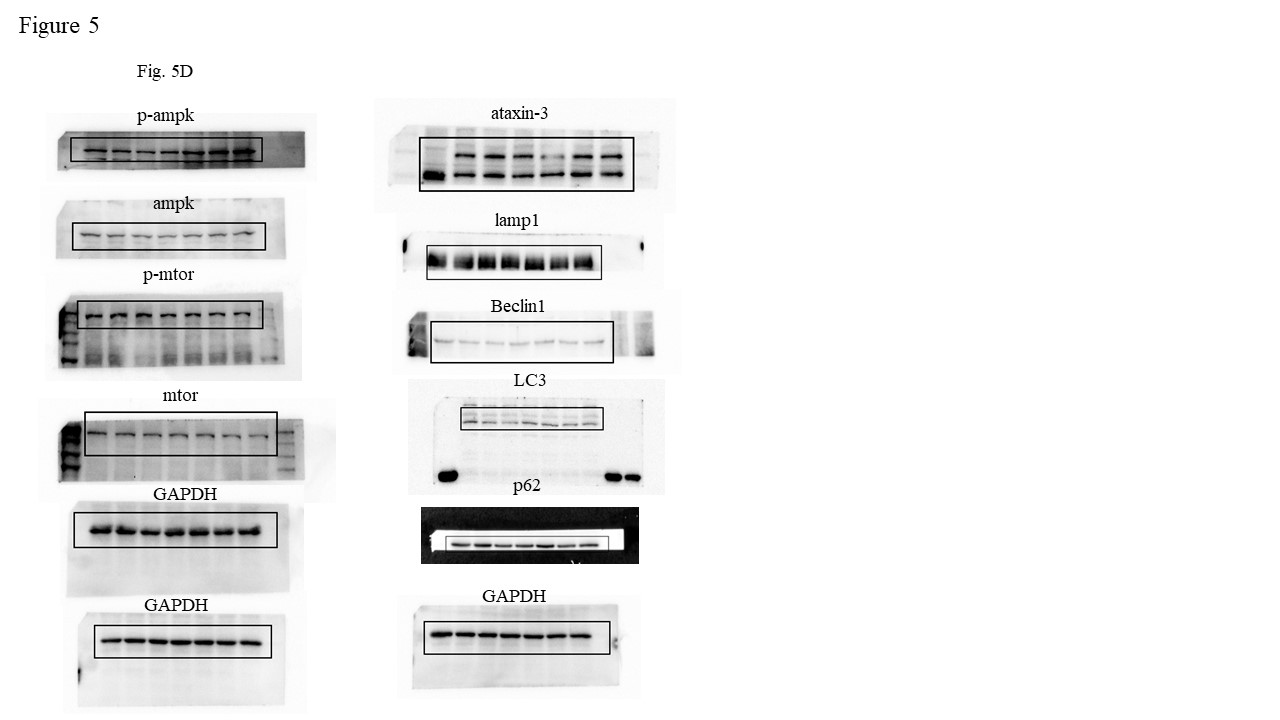


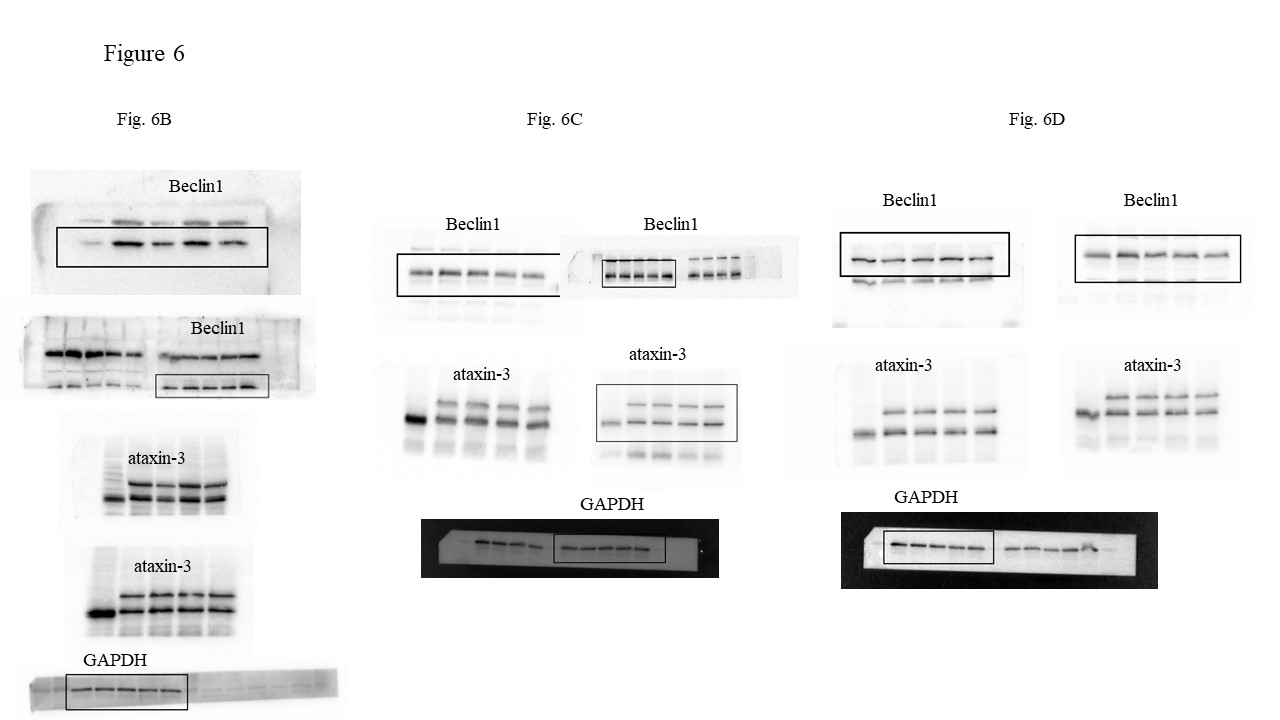


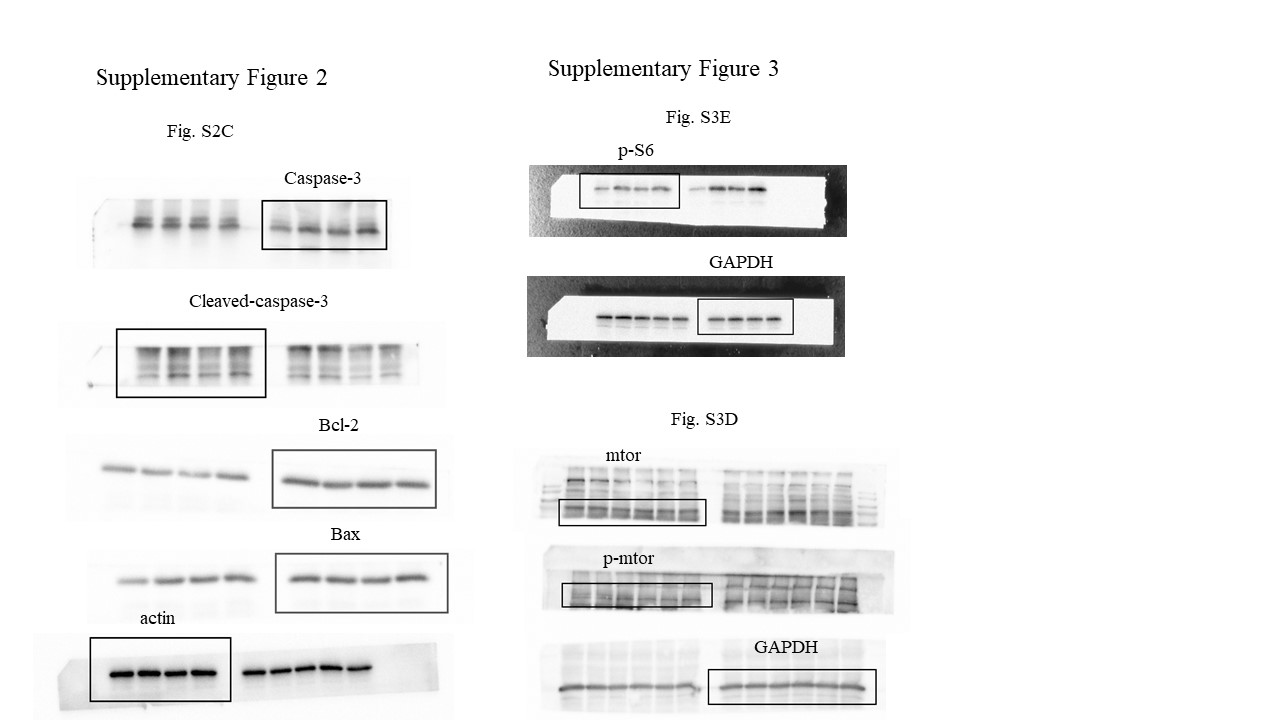

Supplement: Supplementary file 6 — Original Data File [file 41419_2022_5085_MOESM6_ESM.docx]
